# Genome-wide DNA methylation profile changes associated with shell colouration in the Yesso scallop (*Patinopecten yessoensis*) as measured by whole-genome bisulfite sequencing

**DOI:** 10.1186/s12864-021-08055-6

**Published:** 2021-10-14

**Authors:** Changzi Yuan, Junxia Mao, Hongyan Sun, Yiying Wang, Ming Guo, Xubo Wang, Ying Tian, Zhenlin Hao, Jun Ding, Yaqing Chang

**Affiliations:** Key Laboratory of Mariculture & Stock Enhancement in North China’s Sea, Ministry of Agriculture and Rural Affairs, Dalian Ocean University, Dalian, China

**Keywords:** Yesso scallop, DNA methylation, WGBS, Shell colouration, Epigenetic regulation

## Abstract

**Background:**

Mollusca, a phylum of highly rich species, possess vivid shell colours, but the underlying molecular mechanism remains to be elucidated. DNA methylation, one of the most common epigenetic modifications in eukaryotes, is believed to play a vital role in various biological processes. However, analysis of the effects of DNA methylation on shell colouration has rarely been performed in molluscs, limiting the current knowledge of the molecular mechanism of shell colour formation.

**Results:**

In the present study, to reveal the role of epigenetic regulation in shell colouration, WGBS, the “gold standard” of DNA methylation analysis, was first performed on the mantle tissues of Yesso scallops (*Patinopecten yessoensis*) with different shell colours (brown and white), and DNA methylomes at single-base resolution were generated. About 3% of cytosines were methylated in the genome of the Yesso scallop. A slight increase in ^m^CG percentage and methylation level was found in brown scallops. Sequence preference of nearby methylated cytosines differed between high and low methylation level sites and between the brown- and white-shelled scallops. DNA methylation levels varied among the different genomic regions; all the detected regions in the brown group exhibited higher methylation levels than the white group. A total of 41,175 DMRs (differentially methylated regions) were detected between brown and white scallops. GO functions and pathways associated with the biosynthesis of melanin and porphyrins were significantly enriched for DMRs, among which several key shell colour-related genes were identified. Further, different correlations between mRNA expression levels and DNA methylation status were found in these genes, suggesting that DNA methylation regulates shell colouration in the Yesso scallop.

**Conclusions:**

This study provides genome-wide DNA methylation landscapes of Yesso scallops with different shell colours, offering new insights into the epigenetic regulatory mechanism underlying shell colour.

**Supplementary Information:**

The online version contains supplementary material available at 10.1186/s12864-021-08055-6.

## Background

Colour is a common and important trait in many organisms, and is often associated with camouflage, aposematism, mate attraction, thermoregulation and immunity [1–3]. Mollusca is the second-largest phylum of animals on the planet, comprising highly rich species. Molluscs are known for their colourful shells, which have attracted collectors and scientists for hundreds of years. Compared with plants and vertebrates, there is limited knowledge of the molecular mechanism underlying shell colouration in molluscs due to its complexity. Generally, the shell colour is related to the presence of shell pigments, including melanin, carotenoids, porphyrins and bile pigments, which have been identified in some molluscs [4]. Though environmental conditions and food intake can sometimes affect shell pigmentation [5, 6], many breeding studies have demonstrated that the shell colour trait is under genetic control [7–10], and some shell colour-related genetic markers have been detected [11–14]. Benefiting from next-generation sequencing, extensive transcriptome analysis has been performed in molluscan species, and some pathways (such as melanogenesis, the heme biosynthesis pathway and the porphyrin and chlorophyll metabolism pathway) associated with shell colour have been identified [15–21]. However, the underlying regulation mechanism remains to be elucidated.

Epigenetic modification is an important gene regulatory system affecting gene expression without altering nucleotide sequences [22–24]. DNA methylation is one of the most common epigenetic modifications in eukaryotes and is believed to play a vital role in various biological processes, including transcription regulation, embryogenesis, cellular differentiation, genomic imprinting, X-chromosome inactivation, transposon silencing and so on [25–31]. In plants and some vertebrates, DNA methylation is also involved in the regulation of colouration, such as flower colours [32], eggshell colours [33] and animal skin and coat colours [34]; this suggests that it is also likely involved in the regulation of shell colour. However, to date, no published studies have addressed this hypothesis.

Though limited, an increasing number of DNA methylation analyses have been performed in molluscs due to the important regulation role of DNA methylation; however, most have focused on oysters. For example, global DNA methylation analysis by whole-genome bisulfite sequencing (WGBS) in the Pacific oyster *Crassostrea gigas* indicated that DNA methylation plays a complex role in regulating genome activity and is involved in the regulation of development processes and stress and environmental responses [35–38]. In the pearl oyster *Pinctada fucata martensii*, DNA methylomes of biomineralization-related tissues were analysed by methylated DNA immunoprecipitation sequencing (MeDIP-Seq) to explore the regulatory mechanism underlying shell formation [39]. In the Yesso scallop *Patinopecten yessoensis*, dynamic DNA methylation levels were detected during gametogenesis and early development [40]. Further, different DNA methylation levels have been detected in Yesso scallops with different adductor muscle colours using the MethylRAD technique [41]. However, there are few published analyses of the effects of DNA methylation on shell colouration in molluscs, limiting the current knowledge of the molecular mechanism of shell colour formation.

The Yesso scallop (*P. yessoensis*) is a large and old (dating back to ~ 350 Ma) bivalve animal that can be found living along the coastlines of northern Japan, the Russian Far East and northern Korea. It is one of the most important aquaculture shellfish in Asian countries and is consumed worldwide. The shell colour of the Yesso scallop represents a polymorphism whereby most individuals possess brown left valves while a small percentage of individuals have white left valves. Unlike some other molluscs with complex shell colours, the shell colour pattern of the Yesso scallop is unambiguous and relatively simple, making it a good material for shell colour study. Due to the important evolutionary and economic position of the Yesso scallop, much research has been conducted on this species, including genome and transcriptome sequencing [42], epigenetics analyses [14, 40] and genetic breeding for shell colour [41]; these studies provide valuable genetic resources for research on the biological problems of the Yesso scallop. WGBS, which is considered the “gold standard” of DNA methylation analysis, is the most effective method to study genomic methylation patterns; it can generate methylomes at single-base resolution without a preference [43]. In the present study, WGBS analysis was first performed on the mantle tissues of Yesso scallops with two different shell colours (brown and white) in order to generate DNA methylomes at single-base resolution for this species. The general characteristics of DNA methylation in the two shell colour Yesso scallops were analysed and DNA methylation changes between the two kinds of scallops were examined. The aim was to explore the impact of DNA methylation on shell colouration and provide new genome-wide evidence for understanding the epigenetic regulatory mechanism underlying the shell colour trait.

## Results

### Genome-wide DNA methylation landscape in the Yesso scallop

To investigate the overall methylation patterns of the Yesso scallop and detect those sites associated with shell pigmentation, WGBS was performed on the mantle tissues of two different shell colour populations (brown and white) of Yesso scallops. A total of 147.54 Gb and 150.25 Gb raw bases were generated for the two groups, respectively, with an average of 49.18 Gb and 50.08 Gb, respectively, for each replicate, representing > 30 × the reference genome [44]. Detailed description of the sequencing data is provided in Table 1. The raw data have been deposited into the NCBI SRA database with the accession number PRJNA695315.

**Table 1:** Data summary of BS-Seq for the two shell colour Yesso scallops; data represents three replicates

Samples	Raw Reads/Raw Base (Gb)	Clean Reads/Clean Base (Gb)	BS Conversion Rate	Total Mapped Reads	Mapping Rate
Brown 1	345,103,250/51.77	334,652,788/49.86	99.88%	163,955,550	48.99%
Brown 2	316,013,962/47.40	304,170,606/45.26	99.85%	140,235,924	46.10%
Brown 3	322,452,000/48.37	312,721,358/46.63	99.88%	143,072,896	45.75%
White 1	319,764,220/47.96	309,094,520/46.05	99.82%	140,779,852	45.55%
White 2	380,237,074/57.04	366,323,134/54.55	99.92%	176,451,976	48.17%
White 3	301,652,872/45.25	294,587,968/43.97	99.90%	148,013,856	50.24%

**Fig. 1:**
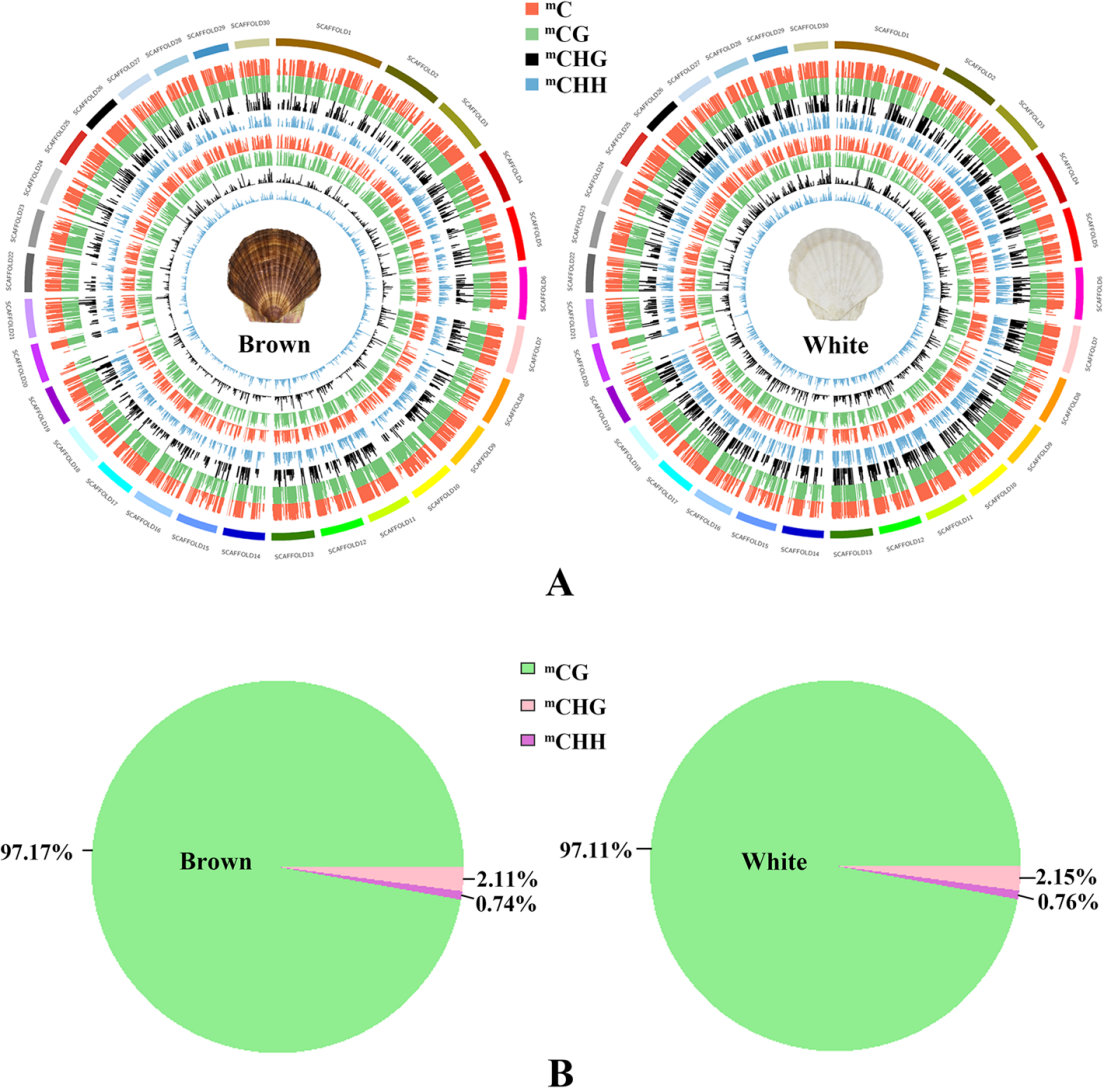
DNA methylation landscape of Yesso scallops with different shell colours. **A** Density plot of 5-methylvytosine in ^m^CG, ^m^CHG and ^m^CHH sequence contexts. The first circle (outermost) represents the 30 longest scaffolds in the genome of the Yesso scallop; circles 2–5 represent the ratios of each sequence context (i.e., ^m^C/covered C, ^m^CG/covered CG, ^m^CHG/covered CHG, ^m^CHH/covered CHH); circles 6–9 represent the average methylation levels of each sequence context. **B** The proportion of ^m^C in the three sequence contexts

The distributions of the DNA methylation sites in the whole genome of the Yesso scallop were separately analysed in the brown and white individuals (Fig. 1a, Table S1). Overall, approximately 3.07 and 2.94% of all genomic C sites were methylated in the brown and white groups, respectively. Methylation was found to exist in three sequence contexts: CG, CHG and CHH (where H represents A, C or T). The genome of the brown group exhibited 24.35% (^m^CG), 0.12% (^m^CHG) and 0.09% (^m^CHH) methylation on the total sequenced CG, CHG and CHH sites, respectively. Accordingly, the white group exhibited 22.71, 0.12 and 0.09% methylation in the CG, CHG and CHH contexts, respectively, with a slight decrease in CG methylation percentage compared with the brown group. The proportions of ^m^CG, ^m^CHG and ^m^CHH on the total ^m^C sites were analysed (Fig. 1b, Table S1), which reflects the distributions of ^m^Cs in the three sequence contexts. Similar rates were found in the brown and white groups, with methylcytosine most often found at CG sites (~ 97%) and much less frequently at CHG (~ 1%) and CHH (~ 2%) sites.

**Fig. 2:**
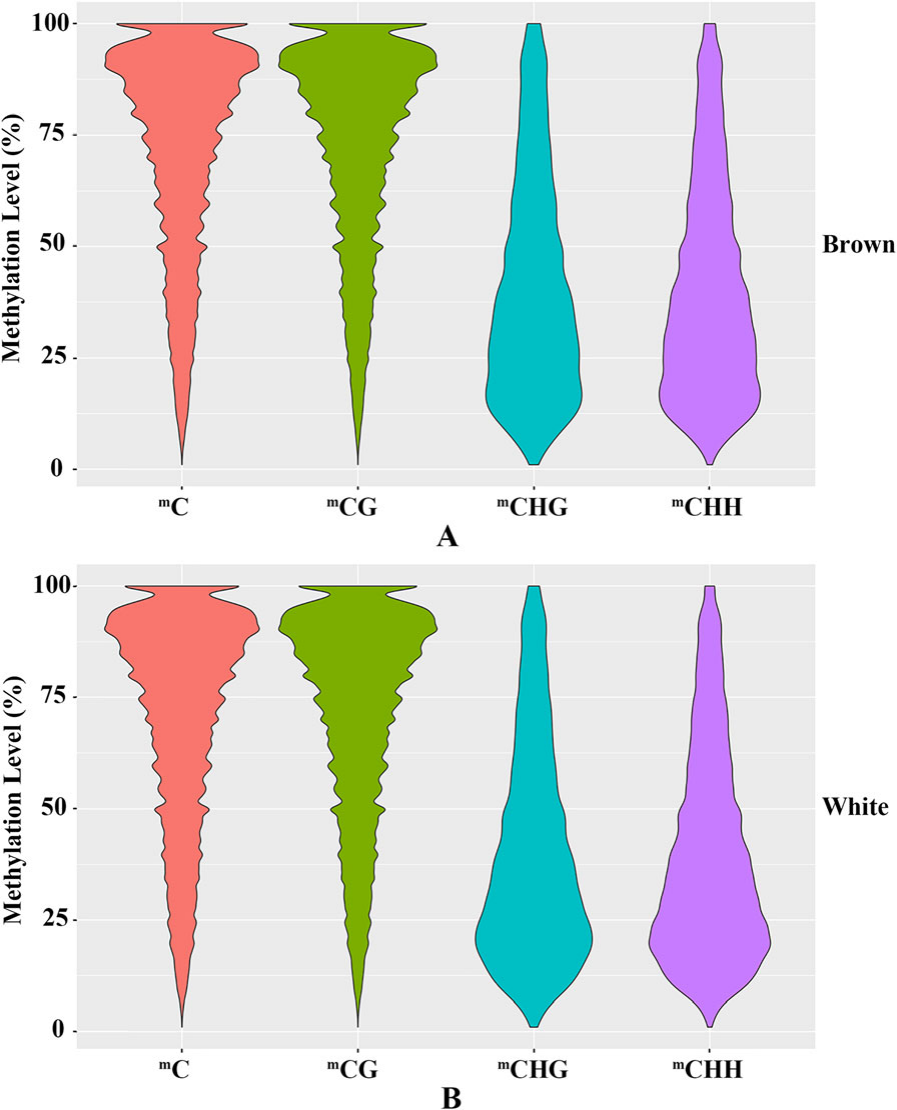
The distribution of DNA methylation levels of all ^m^C sites and different sequence contexts in Yesso scallops with two different shell colours

On the whole, the methylation levels of the two shell colour groups shared similar trends, with high levels in CG sites and low levels in CHG and CHH sites (Fig. 2, Table S1). The average methylation levels in the genome of the brown group were 2.34, 17.96, 0.18 and 0.16% for all detected C, CG, CHG and CHH sites, and 71.33, 72.24, 40.61 and 39.98% for the ^m^C, ^m^CG, ^m^CHG and ^m^CHH sites, respectively. Moreover, the average methylation levels in the genome of the white group were 2.17, 16.39, 0.15 and 0.12% for all detected C, CG, CHG and CHH sites, and 69.94, 70.86, 39.49 and 39.12% for the ^m^C, ^m^CG, ^m^CHG and ^m^CHH sites, respectively. Overall, all types of sites in the white group exhibited slightly decreased methylation levels compared with those of the brown group.

### Sequence preferences analysis for DNA methylation

To explore the sequence preference of methylated sites, Logo Plots [45] were utilized to analyse the sequence information of each methylated site and its nearby regions (a total of 9-mer sequences containing a methylated site). For the CG context, sites with a methylation level > 75% were defined as high methylation level sites and the others were defined as low methylation level sites. For the CHG and CHH contexts, sites with a methylation level > 25% were defined as high methylation level sites and the others were defined as low methylation level sites. The sequence preference around methylated C sites varied among the three types of sequence contexts, and obvious differences were found between the high and low methylation level sites; differences also existed between the two shell colour groups (Supplementary Figure S1). In the CG context, the bases around the high methylation level sites were distributed evenly, while for the low methylation level sites, ^m^C frequently occurred at the A**CG**A sequence for the brown group and A**CG**T sequence for the white group (Figure S1A). In the CHG context, ^m^C in the high methylation level sites often appeared at the A**CAG**A sequence for the brown group and T**CAG**A sequence for the white group, while ^m^C in the low methylation level sites appeared at the A**CTG**A and A**CTG**T sequences for the brown and white groups, respectively (Figure S1B). In the CHH context, ^m^C frequently occurred at the CAT sequence regardless of the high or low methylation level status of the region (Figure S1C); however, differences in the composition of the nearby bases in the CHH context were also observed between the brown and white groups, e.g., cytosines preferred to be methylated at the **CAT**A sequence for all the methylated sites in the brown group, while preference was for the **CAT**T sequence in the white group.

### DNA methylation pattern in different genomic regions

**Fig. 3:**
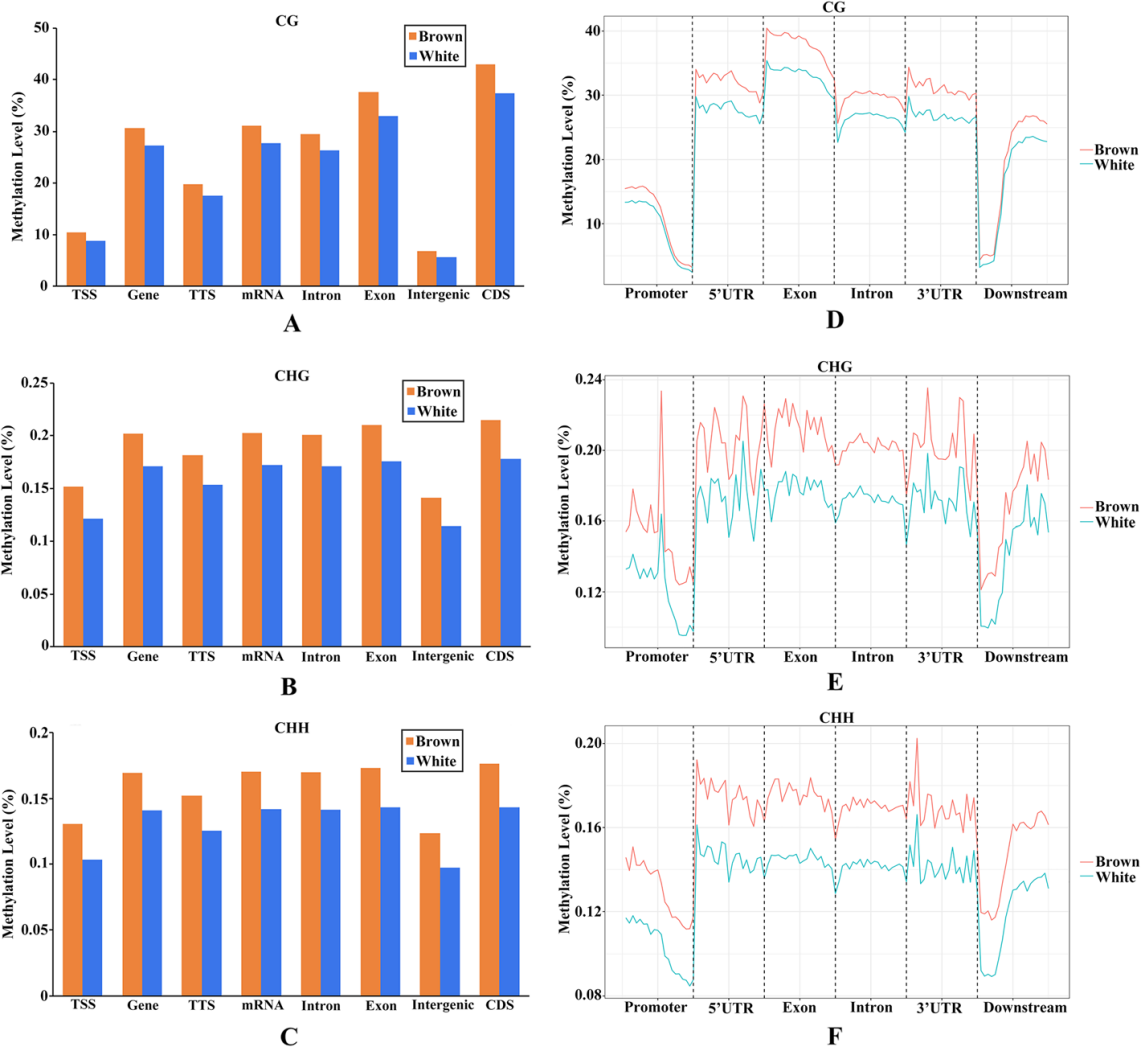
DNA methylation levels in different genomic regions of Yesso scallops with different shell colours. **A**-**C** Distributions of DNA methylation levels on different genome elements separately for the CG, CHG and CHH contexts. **D**-**F** Distributions of DNA methylation levels among gene features separately for the CG, CHG and CHH contexts

To study the DNA methylation profiles of different genomic regions of the Yesso scallop and explore the differences between the brown and white groups, the DNA methylation levels of different genomic regions were compared for each sequence context of the two kinds of scallops. As shown in Fig. 3, DNA methylation levels varied among the different genomic regions; the CG context showed the highest methylation level among the three sequence contexts in each genomic region, while the CHH context showed the lowest. The methylation level of the CG context in both of the shell colour groups was highest in the CDS, followed by the exon, and was lowest in the intergenic regions (Fig. 3a). Similar patterns were found in the other two contexts, but the differences between the different genomic regions were not as obvious as those in the CG context (Fig. 3b and c). The methylation levels within transcribed regions, including the 5’UTR, exon, intron and 3’UTR, and 2 kb upstream (i.e., promoter region) and downstream regions were also evaluated (Fig. 3d, f). In the CG context, the methylation level in the promoter region was the lowest and gradually declined toward the 5’UTR, then rose high in the 5’UTR. 5’UTR and 3’UTR had almost the same methylation levels, with methylation levels slightly higher than the intron regions. The exon regions exhibited the highest methylation level. The methylation level dropped sharply at the start of the 2-kb downstream regions and then rose back (Fig. 3d). A similar methylation pattern was also found in the CHG and CHH contexts, but the average methylation levels within the transcribed regions were comparable (Fig. 3e and f). Moreover, similar methylation patterns were observed in the two shell colour scallops, but all the detected regions in the brown group had higher methylation levels than those in the white group. The differences in the methylation levels of cytosines between the two kinds of scallops may be correlated with shell colouration.

### DMR analysis between the brown and white Yesso scallops

**Fig. 4:**
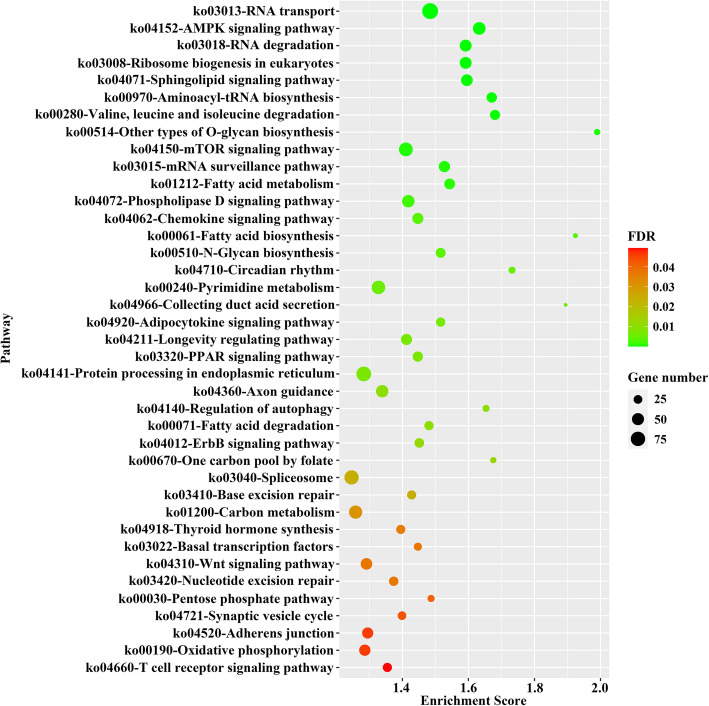
KEGG pathway enrichment analysis of DMRs (ranked by FDR; FDR < 0.05; KEGG pathways got from: https://www.kegg.jp/kegg/kegg1.html)

Differentially methylated regions (DMRs) were examined between the brown and white groups to assess the role of methylation in colouration. A total of 41,175 DMRs were identified (Supplementary Table S2). To analyse the function of these DMRs, GO (Gene Ontology term) and KEGG (Kyoto Encyclopaedia of Genes and Genomes) enrichment analyses were separately performed on the DMR-associated genes. A total of 1132 GO terms were significantly enriched (FDR <  0.05, gene number > 3), covering a variety of functions (Supplementary Table S3). Many of the enriched GO terms were associated with histone modification, such as histone phosphorylation, acetylation, deacetylation, methylation, demethylation and so on; chromosome and DNA structure changes, such as chromatin remodelling, binding and silencing, DNA helicase activity, duplex unwinding and so on; and transcription regulation, such as different transcription factors activities (Table 2). More importantly, several pigmentation-related GO terms were enriched, i.e., pigmentation, melanocyte differentiation, melanosome organization, melanosome transport, endosome to melanosome transport, porphyrin-containing compound biosynthetic process and heme biosynthetic process, which are closely related to melanin and porphyrins formation. KEGG enrichment analysis showed that 39 pathways were significantly enriched at a threshold of FDR <  0.05 and gene number > 3; these pathways were related to RNA biosynthesis, degradation and transport, fatty acid biosynthesis, degradation and metabolism, glycan biosynthesis, carbon metabolism and so on (Fig. 4). It should be noted that the pathway of ‘porphyrin and chlorophyll metabolism’ (KO00860), which was closely associated with shell colour formation, was also significantly enriched with a threshold of *p* <  0.05 (Supplementary Table S4).

**Table 2:** GO enrichment analysis of DMRs

iD	Term	ListHits	FDR (< 0.05)	iD	Term	ListHits	FDR (< 0.05)
**Histone modification**				**Chromosome and DNA structures**			
GO:0033129	positive regulation of histone phosphorylation	4	0.00E+ 00	GO:0006338	chromatin remodeling	28	1.75E-03
GO:0033169	histone H3-K9 demethylation	4	0.00E+ 00	GO:0035562	negative regulation of chromatin binding	4	3.12E-02
GO:0032454	histone demethylase activity (H3-K9 specific)	4	0.00E+ 00	GO:0051304	chromosome separation	4	3.12E-02
GO:0044020	histone methyltransferase activity (H4-R3 specific)	4	0.00E+ 00	GO:0043138	3′-5′ DNA helicase activity	5	0.00E+ 00
GO:0035267	NuA4 histone acetyltransferase complex	15	1.46E-04	GO:0004003	ATP-dependent DNA helicase activity	16	8.62E-04
GO:0018024	histone-lysine N-methyltransferase activity	15	1.46E-04	GO:0032508	DNA duplex unwinding	21	2.79E-03
GO:0043968	histone H2A acetylation	12	3.02E-04	GO:0003678	DNA helicase activity	10	1.54E-02
GO:0000123	histone acetyltransferase complex	15	5.57E-04	GO:0043141	ATP-dependent 5′-3′ DNA helicase activity	8	2.16E-02
GO:0071044	histone mRNA catabolic process	7	2.67E-03	**Transcription regulation**			
GO:0042800	histone methyltransferase activity (H3-K4 specific)	7	2.67E-03	GO:0008023	transcription elongation factor complex	11	0.00E+ 00
GO:0000118	histone deacetylase complex	14	3.16E-03	GO:0033276	transcription factor TFTC complex	7	0.00E+ 00
GO:0051571	positive regulation of histone H3-K4 methylation	6	6.17E-03	GO:0005669	transcription factor TFIID complex	16	2.73E-04
GO:0016575	histone deacetylation	11	8.28E-03	GO:0017053	transcriptional repressor complex	16	8.62E-04
GO:0042826	histone deacetylase binding	28	1.27E-02	GO:0010608	posttranscriptional regulation of gene expression	12	1.36E-03
GO:0070776	MOZ/MORF histone acetyltransferase complex	5	1.41E-02	GO:0006351	transcription, DNA-templated	28	1.75E-03
GO:0017136	NAD-dependent histone deacetylase activity	5	1.41E-02	GO:0000127	transcription factor TFIIIC complex	6	6.17E-03
GO:0043966	histone H3 acetylation	15	1.74E-02	GO:0000976	transcription regulatory region sequence-specific DNA binding	16	1.05E-02
GO:0043967	histone H4 acetylation	11	1.94E-02	GO:0005675	transcription factor TFIIH holo complex	6	2.86E-02
GO:0043981	histone H4-K5 acetylation	8	2.16E-02	**Pigmentation**			
GO:0043982	histone H4-K8 acetylation	8	2.16E-02	GO:0043473	pigmentation	8	6.70E-03
GO:0031065	positive regulation of histone deacetylation	6	2.86E-02	GO:0030318	melanocyte differentiation	7	2.67E-03
GO:0035067	negative regulation of histone acetylation	6	2.86E-02	GO:0032438	melanosome organization	7	0.00E+ 00
GO:0051568	histone H3-K4 methylation	6	2.86E-02	GO:0032402	melanosome transport	6	6.17E-03
GO:0016570	histone modification	4	3.12E-02	GO:0035646	endosome to melanosome transport	7	1.41E-02
GO:0004402	histone acetyltransferase activity	16	3.19E-02	GO:0006779	porphyrin-containing compound biosynthetic process	4	0.00E+ 00
GO:0035064	methylated histone binding	22	3.73E-02	GO:0006783	heme biosynthetic process	12	1.36E-03
GO:0070932	histone H3 deacetylation	7	4.01E-02				
GO:0016573	histone acetylation	12	4.21E-02

**Fig. 5:**
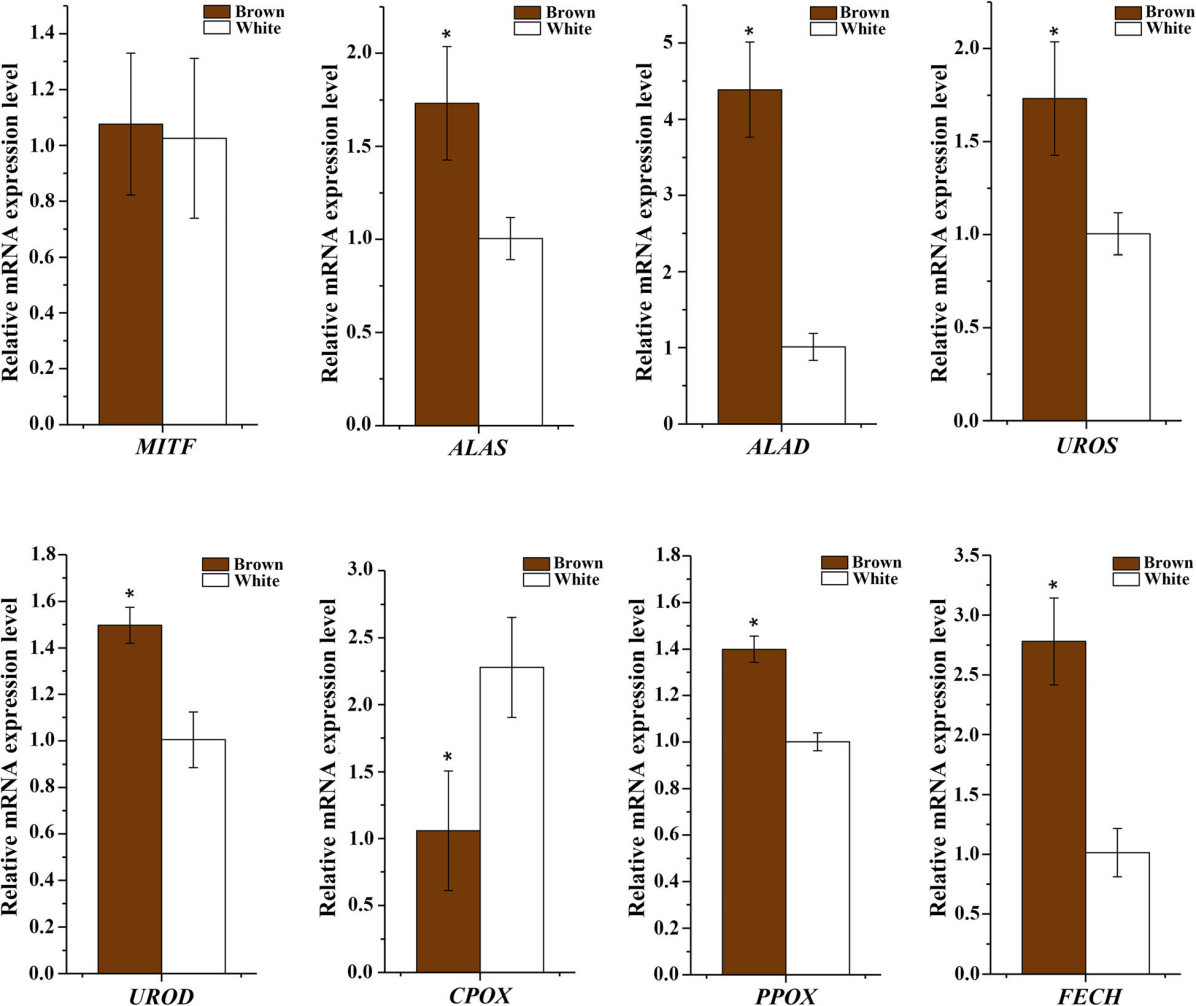
Relative expression levels of shell colour-related genes in the mantle tissues of Yesso scallops with different shell colours. Vertical bars represent the mean ± S.E. (*N* = 3). Asterisks indicate significant differences (*p* < 0.05)

Genes involved in the biosynthesis pathway of melanin and porphyrins were identified among DMRs, and are listed in Table 3. *MITF* (*microphthalmia-associated transcription factor*) is the primary regulator of melanin synthesis. There were three DMRs detected in the gene of *MITF*, and they all exhibited significantly higher methylation levels in the white group than the brown group. Gene expression analysis for the *MITF* gene showed a higher expression level in the brown group than that in the white group, though this difference was not statistically significant (*p* ≥ 0.05) (Fig. 5). The genes *ALAS* (*5-aminolevulinate synthase*), *ALAD* (*delta-aminolevulinic acid dehydratase*), *UROS* (*uroporphyrinogen-III synthase*), *UROD* (*uroporphyrinogen decarboxylase*), *CPOX* (*coproporphyrinogen-III oxidase*), *PPOX* (*protoporphyrinogen oxidase*) and *FECH* (*ferrochelatase*), all from the heme biosynthesis pathway, are involved in the large pathway porphyrin and chlorophyll metabolism. Seven of eight genes in the heme biosynthesis pathway were differentially methylated between the brown and white groups. The methylation levels of all the DMRs in the genes *ALAS*, *UROS* and *PPOX* were higher in the brown group than in the white group; while the methylation levels of DMRs in the *ALAD* and *CPOX* genes were lower in the brown group than the white group; and different methylation sites in the *UROD* and *FECH* genes showed different methylation level changes between the two shell colour groups. Gene expression analysis for the above seven genes showed that aside from *CPOX*, which had a significantly higher expression level in the white group (*p* < 0.05), the other six genes had significantly higher expression levels in the brown group than the white (*p* < 0.05) (Fig. 5).

**Table 3:** DMRs involved in genes associated with shell colouration

Gene Name	Accession ID	Scaffold ID	Position Start	Position End	Methylation level-Brown (%)	Methylation level-White (%)	Methylation Difference (%)	***P*** value (≤0.05)
***ALAS***	LOC110452629	NW_018406720.1	745,001	746,000	80	67.57	12.43	1.98E-02
***ALAD***	LOC110458627	NW_018408167.1	103,001	104,000	22.75	34.55	−11.81	2.04E-03
***UROS***	LOC110449792	NW_018405737.1	1,396,001	1,397,000	15.47	0.24	15.23	4.54E-55
			1,397,001	1,398,000	19.29	0.76	18.53	1.39E-50
			1,398,001	1,399,000	31.86	1.93	29.93	6.29E-79
			1,399,001	1,400,000	76.19	56.57	19.63	1.79E-22
			1,400,001	1,401,000	62.85	39.42	23.44	5.99E-21
			1,401,001	1,402,000	54.34	40.18	14.16	1.63E-06
			1,426,001	1,427,000	56.66	42.68	13.98	7.03E-05
			1,430,001	1,431,000	73.93	59.91	14.02	6.10E-05
***UROD***	LOC110452530	NW_018406690.1	1,375,001	1,376,000	58.46	74.66	−16.2	1.43E-02
			1,378,001	1,379,000	70.97	59.82	11.15	4.75E-02
***CPOX***	LOC110453297	NW_018406955.1	618,001	619,000	66.39	78.23	−11.84	2.89E-04
***PPOX***	LOC110452239	NW_018406583.1	28,001	29,000	71.48	0	71.48	5.79E-07
			31,001	32,000	76.35	64.29	12.07	1.01E-02
***FECH***	LOC110449109	NW_018405575.1	179,001	180,000	71.13	89.35	−18.22	3.47E-05
			183,001	184,000	27.14	13.14	14	4.77E-07
***MITF***	LOC110457295	NW_018407865.1	121,001	122,000	30.3	60.87	−30.57	3.02E-02
			129,001	130,000	33.98	51	−17.02	9.05E-17
			138,001	139,000	38.88	49.16	−10.28	7.10E-03

## Discussion

DNA methylation is one of the most important epigenetic modifications in eukaryotes, but little is known about its role in shell colour formation. In the present study, genome-wide DNA methylation analysis was first performed on the mantle tissues of Yesso scallops with different shell colours using WGBS. The aim of this study was to explore the potential epigenetic regulatory mechanism of the shell colour trait.

DNA methylation is evolutionarily conserved, but markedly different methylation patterns exist among kingdoms [46]. Generally, vertebrates exhibit high DNA methylation levels while the genomes of invertebrates are relatively less methylated [47]. For example, approximately 4–6% of genomic cytosines are methylated in humans [48, 49], and a similar ratio was also found in a marine organism sea squirt from Urochordata (4.07%) [46]; however, quite a small percentage of cytosines (~ 0.1–0.2%) are methylated in the whole genomes of some insects [50, 51, 52]. In the limited available studies, different methylation levels have been found in marine invertebrates; for example, ~ 1.4% of genomic cytosines are methylated in the sea anemone [47], while ~ 3.5% were detected in the sea cucumber [53]. Studies of molluscs have primarily focused on oysters. In the present study, about 3% of genomic cytosines were found to be methylated in the Yesso scallop, slightly higher than that in the Pacific oyster (~ 1.95%) [38] and the Eastern oyster (~ 2.7%) [54]. Relatively low mapping rate of BS-seq sequences was found in the present study (~ 45.5–50.2%), which was probably due to the relatively high polymorphism of molluscan species or sequence differences between the studied individuals and the reference genome. Similar results have also been found in Pacific oyster, such as ~ 48.4–49.3% mapping rate for the BS-seq sequences of the wild Pacific oyster by Wang et al. [38], and ~ 53% in the study by Olson and Roberts [37].

Few studies investigating the function of DNA methylation in shell colouration of molluscs have been carried out. In the current study, whole-genome methylation patterns and levels of the Yesso scallop were first analysed and compared between brown and white shell colour individuals. Overall, the DNA methylation landscapes of the two groups were similar, but slight increases in the ^m^CG percentage and methylation level were detected in the brown group. The sequence preference of methylated cytosines is not always consistent among different species [48, 55]. In the present study, the nearby sequences of the ^m^CG, ^m^CHG and ^m^CHH contexts varied across different methylation level sites (high and low), and differences were also observed between the two shell colour groups. DNA methylation levels varied among different genomic regions, with the highest in CDS; this is similar to the analysis in the Pacific oyster by Wang et al. [38]. In the transcribed regions, exons showed higher methylation levels than introns, consistent with the result observed in the sea cucumber [53], but different to most other species including plants [55, 56], mammals [48, 49] and also some molluscs, such as the Pacific oyster [36], eastern oyster [54] and pearl oyster [39], which showed higher methylation levels in introns. Further, all detected regions in the brown individuals exhibited higher methylation levels than those in the white group. All of the above DNA methylation differences between the two shell colour groups may reflect a mechanism that regulates the process of shell colour formation.

To further assess the function of DNA methylation in shell colouration, DMRs were analysed between the brown and white Yesso scallops. The results suggested that DNA methylation alterations could regulate the formation or transportation processes of melanin and porphyrins, thereby affecting shell colouration. Melanin is one of the most common pigments in animals, and has been identified in many molluscan species, including the Yesso scallop [4, 57]. In the present study, many melanin-related GO functions were significantly enriched in the DMRs between the brown and white Yesso scallops, which indicates that DNA methylation plays an important role in regulating the biosynthesis of shell melanin. *MITF* is a key regulation gene in the melanogenesis pathway [17, 17, 58, 59]. The study by Sun et al. detected a significantly higher expression level of *MITF* in brown Yesso scallops compared to white individuals [17]. Though not statistically significant, a higher expression level of *MITF* was found in the brown scallops in the present study, and a negative correlation was observed between the mRNA expression and DNA methylation levels of the *MITF* gene. Similar findings have also been reported in a study of duck (*Anas platyrhynchos*) plumage colouration, where the DNA methylation level was negatively correlated with the mRNA expression of *MITF-M* [60]. Thus, the results of the present study suggest that the expression of *MITF* could be negatively regulated by DNA methylation, thereby affecting the biosynthesis of shell melanin.

Porphyrin is another pigment in molluscan shells, and has also been found in some gastropods and bivalves [4, 61, 62]. In vertebrates, porphyrins are synthesized as by-products via an evolutionarily ancient heme biosynthesis pathway, which includes eight genes controlling eight enzymatic reactions [63, 64]. The pathway has also been identified in some molluscan species, such as marine snails *Clanculus margaritarius* and *C. pharaonius*, and has been shown to be related to shell colouration [18]. Transcriptome analyses in the Manila clam (*Ruditapes philippinarum*) [20], hard clam (*Mercenaria mercenaria*) [21] and Yesso scallop [15, 19] have identified that the ‘porphyrin and chlorophyll metabolism’ (KO00860) pathway, which is a large pathway containing the heme biosynthesis pathway, is involved in shell colour formation. In the present study, GO functions, including porphyrin and heme biosynthetic processes, and the porphyrin and chlorophyll metabolism pathway were also significantly enriched in the DMRs between the two shell colour scallops, indicating that the biosynthesis process of porphyrins may be regulated by DNA methylation. Seven genes involved in this process were found to be differentially methylated, but complex methylation level changes were observed. Specifically, there were positive correlations between mRNA expression levels and DNA methylation status for the *ALAS*, *UROS*, *CPOX* and *PPOX* genes, a negative correlation was detected for the *ALAD* gene, while different correlations were observed for different DNA methylation sites in the *UROD* and *FECH* genes. These results probably indicate that different DNA methylation modulation mechanisms exist for different genes involved in shell colouration. Further comprehensive studies are needed to verify this assertion.

## Conclusions

In conclusion, WGBS analysis was first performed in Yesso scallops with different shell colours, and DNA methylomes at single-base resolution were generated. Characteristics of the DNA methylation patterns and levels of the Yesso scallop were analysed and compared between the two shell colour groups. DMR analysis between the two kinds of scallops indicated that DNA methylation regulates genes and pathways involved in melanin and porphyrin formation, thereby affecting shell colouration. Moreover, different correlations between mRNA expression levels and DNA methylation status were found in different shell colour-related genes. This study provides new genome-wide evidence for understanding the epigenetic regulatory mechanism underlying the shell colour trait.

## Materials and methods

### Scallop collection

Two-year-old Yesso scallops with brown shell colors were collected from the Dalian Zhangzidao Sea area (Liaoning, China). The scallops were acclimated in the laboratory for 1 week and then sampled following the procesures in [65]. Briefly, filtered and aerated seawater was maintained at approximately 8 °C, which is within the optimum temperature range for their growth. The mantle tissues of the left valves were sampled for DNA extraction. The tissues were immediately frozen in liquid nitrogen and stored in an ultra-low temperature freezer (− 80 °C). To avoid the effect of sex on DNA methylation, scallops with the same sex (male) were chosen in the present study. No specific permits were needed for the described field studies as the Yesso scallops utilized in this study were cultured marine animals provided for market sale, and they are not part of an endangered or protected species. All experiments were conducted following the regulations of the local and central government.

### DNA extraction, library construction and sequencing

Genomic DNA of the mantle tissues was extracted using a standard phenol-chloroform method. The DNA quality and purity were tested by 1% agarose gel electrophoresis and the NanoDrop2000 spectrophotometer (Thermo Scientific, Wilmington, DE, USA). A total of 5.2 μg genomic DNA spiked with 26 ng lambda DNA was fragmented to 200 ~ 300 bp by sonication with Covaris S220 Focused-ultra sonicator (Covaris, Woburn, MA, USA). Lambda DNA was used as an unmethylated control for calculating the bisulphite conversion rate. End-repair, 3′-end dA addition and cytosine-methylated adaptor ligation were subsequently performed. Then, the DNA fragments were treated twice with bisulphite by the EN DNA methylation Gold™ Kit (Zymo Research, Irvine, CA, USA). Through bisulphite treatment, unmethylated C changed to U while methylated C kept unchanged. The libraries were constructed using TruSeq DNA Methylation Kit (Illumina, San Diego, CA, USA) according to the manufacturer’s instructions. Finally, all the libraries were subjected to 150 bp paired-end sequencing on the Illumina Hiseq X TEN genomic sequencing platform. Three biological replicates separately for each kind of shell color scallops were utilized for WGBS libraries construction and sequencing.

### Sequencing data analysis

Raw sequencing data was processed using fastp software [66] to remove reads containing adapters or ambiguous “N” nucleotides and low-quality reads. The remaining high-quality clean reads were used for the subsequent analyses. Then, the clean reads were aligned to the Yesso scallop genome [44] using Bismark software [67] with the default parameters. Conversions of C to T and G to A were separately made in the reference genome and sequence reads before alignment, and the bisulfite-converted genome was indexed by Bowtie2 [68]. The base distribution nearby the methylated cytosine was analyzed by Seqlogo [45], including the frequency of different methylated cytosine sequence contexts, the Logo Plots for 9-mer sequence containing the methylated cytosine, and sequence features of different sequence contexts.

Methylation sites were identified using MethylKit software [69] with the settings that at least 10 reads covering a site (Covered C) and at last one read supported methylated C. Binomial test was used to determine a real methylation site with the FDR ≤ 0.05. Differentially methylated regions (DMRs) were subsequently identified by MethylKit with an unadjusted *p* value threshold of 0.05 and an absolute cutoff of 10% between the two shell color groups. GO and KEGG pathway (https://www.kegg.jp/kegg/kegg1.html) enrichment analysis of DMR related genes were performed respectively using R software based on the hypergeometric distribution algorithm with the following formulas:

$$ p=1-\sum \limits_{f-0}^{m=1}\frac{\left(\begin{array}{l}M\\ {}i\end{array}\right)\left(\begin{array}{l}N-M\\ {}n-i\end{array}\right)}{\left(\begin{array}{l}N\\ {}n\end{array}\right)} $$

$$ Enrichment=\raisebox{1ex}{$\frac{m}{n}$}\!\left/ \!\raisebox{-1ex}{$\frac{M}{N}$}\right. $$

where N is the number of all genes with GO or KEGG annotations, n is the number of genes related to DMR involved in N, M is the number of genes with a specific GO or KEGG annotation, and m is the number of genes related to DMR involved in M. GO functions or pathways with the FDR lower than 0.05 were considered to be significantly enriched.

### Quantitative real-time PCR analysis

qRT-PCR analysis was carried out mainly according to the method mentioned in [65]. Total RNA was isolated from each sample using an RNAprep pure tissue kit (Tiangen). The quantity and quality of total RNA were determined by using the Micro-Spectrophotometer NV3000 (Vastech) and agarose gel electrophoresis. First strand cDNA was synthesized using PrimeScript RT reagent Kit (TaKaRa) following the manufacturer’s protocol. All of the cDNA products were diluted to 200 ng/ul for use as the templates in quantitative real-time PCR. qRT-PCR was conducted using the FastStart Essential DNA Green Master kit (Roche) on a Roche Light Cycler 96 System (Roche) The running program was as follows: 95 °C for 10 min, followed by 40 cycles of 95 °C for 10 s and 60 °C for 30 s, then 95 °C for 10 s and 65 °C for 1 min, finally 97 °C for 1 s and 37 °C for 30 s. The *β-actin* gene was selected as a reference gene. All the primers used for qRT-PCR were designed by Primer Premier 5.0 software, and the primer sequences and products length were listed in Supplementary Table S5. The specificity of the primers was assessed by alignment with the Yesso scallop genome by BLASTN with an e-value of 1e-10. Melting curve analysis was also performed to verify that each primer set amplified a single product. Three technical replicates were performed for each reaction and three individuals for each type of shell color strain were used as biological replicates.

The data from real-time PCR was analysed by the 2^-ΔΔCT^ method [70] to evaluate the relative gene expression levels of the mantle tissues in brown and white Yesso scallops. Statistical analysis of the data was performed with SPSS (version 19.0) software using the independent T-Test. Differences were considered significant at *p* < 0.05.

## Supplementary Information

**Additional file 1: Figure S1.** Sequence preferences for DNA methylation in different sequence contexts in two shell colour Yesso scallops. A. Sequence preference analysis in CG context. For this type, sites with methylation level >75% were defined as high methylation level sites and others were low methylation level sites. B. Sequence preference analysis in CHG context. C. Sequence preferences in CHH context. For CHG and CHH context, sites with methylation level >25% were defined as high methylation level sites and others were low methylation level sites. **Table S1.** Statistical results of methylated cytosines in different sequence contexts. **Table S5.** Primers for quantitative real-time PCR used in this study.

**Additional file 2: Table S2.** DMRs detected between the brown and white Yesso scallops. **Table S3.** All GO terms significantly enriched for differentially methylated regions. **Table S4.** All KEGG pathways significantly enriched for differentially methylated regions (*p* < 0.05).

## Data Availability

The raw data of WGBS have been deposited into the NCBI SRA database with the accession number of PRJNA695315. The datasets generated during this study are included in the article and its supplementary information files.

## Abbreviations

WGBS: Whole-genome bisulfite sequencing
DMR: Differentially methylated region
*MITF*: *Microphthalmia-associated transcription factor*
*ALAS*: *5-aminolevulinate synthase*
*ALAD*: *Delta-aminolevulinic acid dehydratase*
*UROS*: *Uroporphyrinogen-III synthase*
*UROD*: *Uroporphyrinogen decarboxylase*
*CPOX*: *Coproporphyrinogen-III oxidase*
*PPOX*: *Protoporphyrinogen oxidase*
*FECH*: *Ferrochelatase*
GO: Gene Ontology term
KEGG: Kyoto Encyclopedia of Genes and Genomes
