# Supplementary material for: Genome-wide DNA methylation profile changes associated with shell colouration in the Yesso scallop (Patinopecten yessoensis) as measured by whole-genome bisulfite sequencing
Source: BMC Genomics. 2021 Oct 14;22:740. doi: 10.1186/s12864-021-08055-6 (PMC8515700; doi:10.1186/s12864-021-08055-6)
Supplement: Supplementary file 1 — Additional file 1: Figure S1. Sequence preferences for DNA methylation in different sequence contexts in two shell colour Yesso scallops. A. Sequence preference analysis in CG context. For this type, sites with methylation level >75% were defined as high methylation level sites and others were low methylation level sites. B. Sequence preference analysis in CHG context. C. Sequence preferences in CHH context. For CHG and CHH context, sites with methylation level >25% were defined as high methylation level sites and others were low methylation level sites. Table S1. Statistical results of methylated cytosines in different sequence contexts. Table S5. Primers for quantitative real-time PCR used in this study. [file 12864_2021_8055_MOESM1_ESM.docx]

Supplementary Figures:


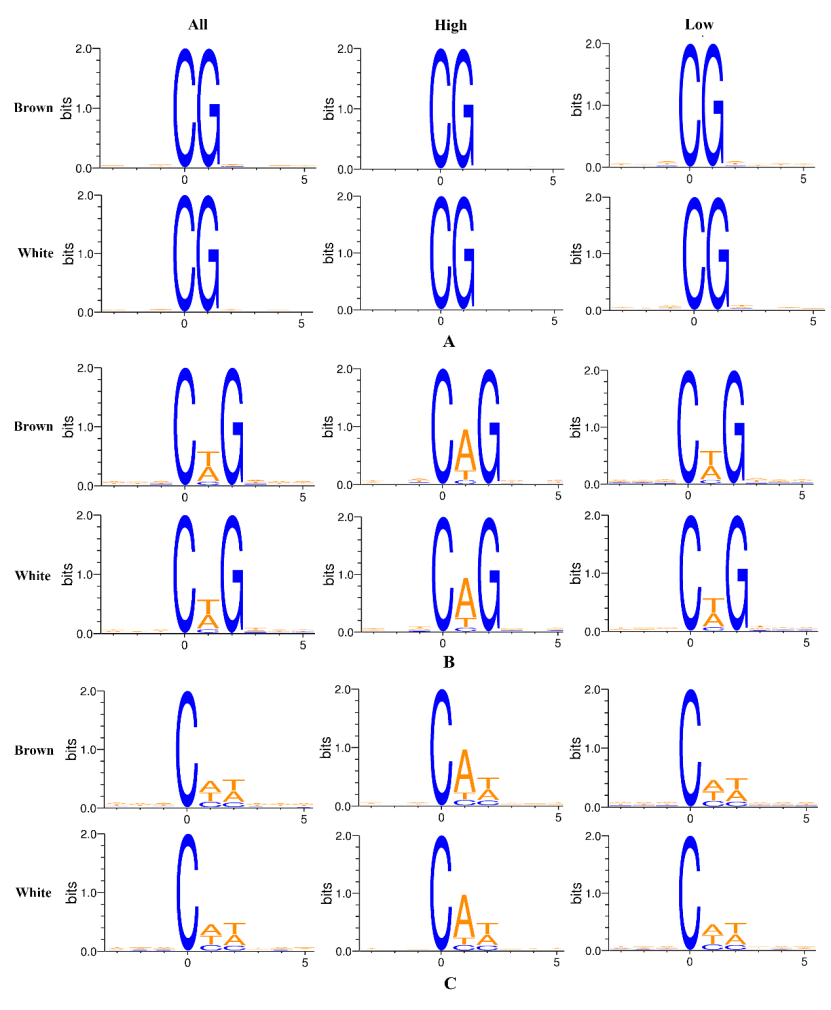
Figure S1. Sequence preferences for DNA methylation in different sequence contexts in two shell color Yesso scallops. A. Sequence preference analysis in CG context. For this type, sites with methylation level >75% were defined as high methylation level sites and others were low methylation level sites. B. Sequence preference analysis in CHG context. C. Sequence preferences in CHH context. For CHG and CHH context, sites with methylation level >25% were defined as high methylation level sites and others were low methylation level sites.

Supplementary Tables:

Table S1. Statistical results of methylated cytosines in different sequence contests.

| Sample | Context | Covered C | ^m^C | ^m^C Percent (%) | Covered C Methyl Level (%) | ^m^C Methyl Level (%) |
| --- | --- | --- | --- | --- | --- | --- |
| Brown | CG | 19,955,059 | 4,859,256 | 24.35 | 17.96 | 72.24 |
|  | CHG | 28,971,528 | 36,643 | 0.13 | 0.18 | 40.61 |
|  | CHH | 113,725,596 | 105,234 | 0.09 | 0.15 | 39.98 |
|  | C | 162,652,183 | 5,001,133 | 3.07 | 2.34 | 71.33 |
| White | CG | 25,847,550 | 5,868,813 | 22.71 | 16.39 | 70.86 |
|  | CHG | 36,973,922 | 45,563 | 0.12 | 0.15 | 39.49 |
|  | CHH | 142,992,091 | 129,658 | 0.09 | 0.12 | 39.12 |
|  | C | 205,813,563 | 6,044,034 | 2.94 | 2.17 | 69.94 |

Table S5. Primers for quantitative real-time PCR used in this study.

| Name | Primer sequences (5’-3’) | Note |
| --- | --- | --- |
| *ALAS-*F | TCAAGACTGGCTCGCTAT | qRT-PCR |
| *ALAS-*R | CGACTCAGGAACGGACAT | qRT-PCR |
| *ALAD-*F | AAGTTCCCTTATGTAGACC | qRT-PCR |
| *ALAD-*R | CCAGTGACAAGTAGTGCC | qRT-PCR |
| *UROS-*F | AGGCATTGTGATGAAGTT | qRT-PCR |
| *UROS-*R | TCCCGTTATGTCTCCTAT | qRT-PCR |
| *UROD-*F | AAAGGTTGAGCCTGTCCC | qRT-PCR |
| *UROD-*R | GCACCCAATAACCGTCTT | qRT-PCR |
| *CPOX-*F | ATCCCAACATTCCAACAA | qRT-PCR |
| *CPOX-*R | ATCCAGGTAAGAAGGTGT | qRT-PCR |
| *PPOX-*F | TGGGTGACGCATTTGTTA | qRT-PCR |
| *PPOX-*R | GAAGGATTGGGTGGCTCT | qRT-PCR |
| *FECH-*F | TTATGTCGCCATTCCAGC | qRT-PCR |
| *FECH-*R | CATCAGGTCACGGTCAAG | qRT-PCR |
| *β-actin-*F | CCAAAGCCAACAGGGAAAAG | qRT-PCR |
| *β-actin-*R | TAGATGGGGACGGTGTGAGTG | qRT-PCR |
